# Effectiveness and durability of Interceptor® long-lasting insecticidal nets in a malaria endemic area of central India

**DOI:** 10.1186/1475-2875-11-189

**Published:** 2012-06-10

**Authors:** Rajendra M Bhatt, Shri N Sharma, Sreehari Uragayala, Aditya P Dash, Raghavendra Kamaraju

**Affiliations:** 1 National Institute of Malaria Research Field Unit, Raipur, 492015, India; 2 National Institute of Malaria Research, Sector 8, Dwarka, New Delhi-110 077, India; 3 World Health Organization, Southeast Asia Regional Office, New Delhi, India

**Keywords:** *Anopheles culicifacies*, Bioassay, Density, Interceptor nets, Malaria, Mosquitoes

## Abstract

**Background:**

In the present study, Interceptor®, long-lasting polyester net, 75 denier and bursting strength of minimum 250 kPa coated with alpha-cypermethrin @ 200 mg/m^2^ was evaluated for its efficacy in reducing the mosquito density, blood feeding inhibition and malaria incidence in a tribal dominated malaria endemic area in Chhattisgarh state, central India. Its durability, washing practices and usage pattern by the community was also assessed up to a period of three years.

**Methods:**

The study was carried out in two phases. In the first phase (September 2006 to August 2007), 16 malaria endemic villages in district Kanker were randomized into three groups, viz. Interceptor net (LN), untreated polyester net (100 denier) and without net. Malaria cases were detected by undertaking fortnightly surveillance by home visits and treated as per the national drug policy. Mosquito collections were made by hand catch and pyrethrum space spray methods from human dwellings once every month. Slide positivity rate (SPR) and malaria incidence per 1000 population (PI) were compared between the three study arms to assess the impact of use of Interceptor nets. Simultaneously, wash resistance studies were carried out in the laboratory by doing cone bioassays on Interceptor LNs washed up to 20 times. Activities undertaken in second Phase (April 2008 to October 2009) after an interval of about 18 months post-net distribution included questionnaire based surveys at every six months, i.e. 18, 24, 30 and 36 months to observe durability, usage pattern of LNs and washing practices by the community. After 36 months of field use, 30 nets were retrieved and sampled destructively for chemical analysis.

**Results:**

Interceptor nets were found effective in reducing the density, parity rate and blood feeding success rate of main malaria vector *Anopheles culicifacies* as compared to that in untreated net and no net villages. SPR in LN villages was 3.7% as compared to 6.5% in untreated and 11% in no net villages. PI in LN villages was 16.4 in comparison to 24.8 and 44.2 in untreated polyester net and no net villages respectively. In surveys carried out after three years of initial distribution, 78.7% (737/936) nets were still in possession with the households, of which 68% were used every night. *An. culicifacies* mortality was >80% in cone bioassays done on LNs washed up to 20 times in laboratory. Mean alpha-cypermethrin content was 43.5 ± 31.7 mg/m^2^ on Interceptor LNs withdrawn after three years of household use against the baseline specification of 200 mg/m^2^. A gradual increase in the proportion of holed nets was observed with the increased period of usage.

**Conclusion:**

Interceptor nets were highly effective in reducing vector densities as well as malaria incidence in the study villages. Availability of 78% nets with the households in usable condition clearly indicated durability of Interceptor LNs up to three years in the rural setting of India. The nets were found to contain an effective concentration of alpha-cypermethrin against malaria vector after three years of household use.

## Background

Long-lasting insecticidal nets (LNs) are being widely promoted for malaria vector control in malaria endemic countries [1]. The use of LNs is an effective intervention method with an efficacy lasting about three to five years. The World Health Organization has given full recommendation to Olyset®, PermaNet® 2.0 and Yorkool® LNs, while interim recommendation has been given to Interceptor® and several other LN brands [2].

The Interceptor® LN is manufactured by M/s. Sunshine World Net 2003 Co. Ltd. Ratchaburi, Thailand, under the license of M/s. BASF Agro B.V. Arnhem (NL) Wädenswil Branch, BP 69, CH-8820 Wädenswil, Switzerland and distributed by BASF. The specifications of Interceptor nets used in the present study were 100% polyester, multifilament yarn: 75 denier with a minimum 250 kPa bursting strength, mesh: 24 holes/cm^2^; density: 30 ± 2 g/m^2^; active ingredient: 200 mg/m^2^ alpha-cypermethrin coated on polyester fibers. The nets used measured 180 cm in length, 160 cm width and 150 cm height, and had a total surface area of 13.92 m². WHOPES supervised laboratory and field trials have demonstrated wash resistance and efficacy of Interceptor nets [3]. Field trials carried out with alpha-cypermethrin treated bed nets have shown promising results in terms of efficacy against mosquito vectors and malaria incidence in India [4,5].

Various field trials have shown further evidence that Interceptor nets are effective against malaria vectors in different countries or settings [6-10]. WHOPES has made interim recommendations for use of Interceptor nets for prevention and control of malaria based on a Phase II study, and requires large-scale field studies for making full recommendations for the use of these nets in the national malaria control programme.

Chhattisgarh state in India is endemic for malaria. The hilly forested and tribal dominated districts, 5 in the north and 3 in the south including Kanker, contribute >90% of the reported malaria cases. In 2010, the state ranked third by reporting about 10% of the total malaria cases and 14% of *Plasmodium falciparum* cases in the country [11]. *Anopheles culicifacies* is the main malaria vector. Malaria continues to be a major health problem especially in difficult to reach areas. In view of this, an evaluation of Interceptor LNs was undertaken in district Kanker to assess the impact on vector density, malaria incidence, bio-efficacy and durability over a three-year period. While plenty of scientific evidence shows effectiveness of treated nets, little is known about durability of various brands of long-lasting insecticidal nets under operational conditions of use and therefore this element of the study makes it important and highly relevant to the national malaria control programme.

## Methods

### Study area

**Figure 1:**
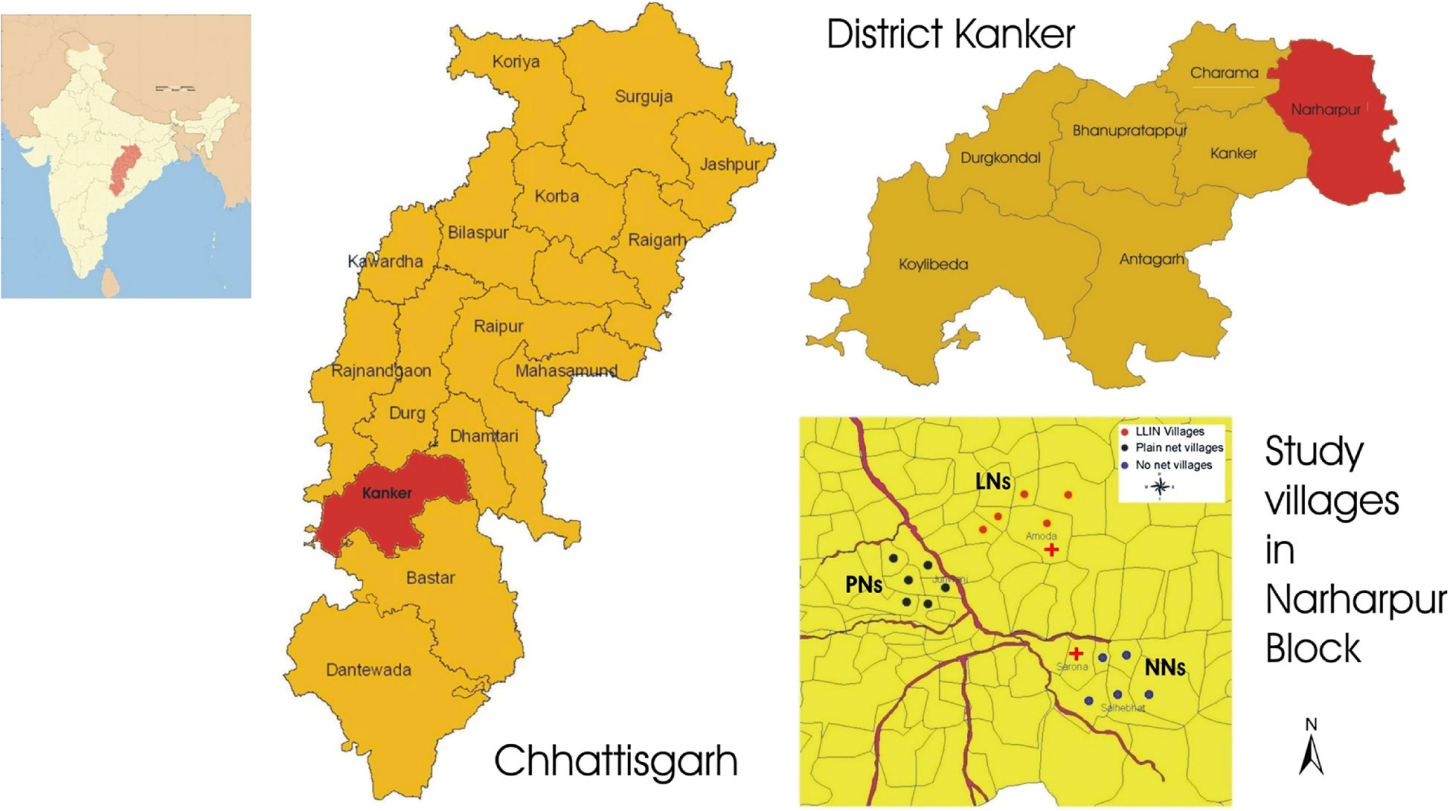
The map showing the location of study villages.

The study was undertaken during September 2006 to August 2007 in 16 villages of district Kanker in Chhattisgarh state central India (Figure 1). Durability surveys were carried out after 18, 24, 30 and 36 months of net distribution (April 2008 to October 2009). District Kanker (pop. 763,549) is one of the malaria endemic districts in the state. It has an area of 5,285 km^2^ lying between latitudes 19°09’ and 20°06’ North and longitudes 80°30’ to 81°15’ East. About 60% of the area is hilly and forested. The district has a tropical climate with an average annual rainfall of 1,100 mm received between June and October in 60–70 rainy days. The villages are scattered and mostly inhabited by tribals belonging to Gond tribe of marginal socio-economic status. The houses are brick built with tiled or thatched roof and mud plastered walls and flooring. There are about 4–5 bedrooms including 1–2 separate rooms for cattle and fodder within the compound. Mud plastering is done generally for about six times in a year or more due to festivals and family occasions or celebrations. People generally sleep in rooms during all seasons and quite often the elder members sleep in verandah within the compound. Subsistence agriculture is the main occupation and rain-fed paddy is the principal crop. The Annual Parasite Index (API) of the district during 2002 to 2006 ranged from 23.5–39.1 cases/1,000 population per year. Proportion of *P. falciparum* infections during the corresponding period ranged between 89.4 and 95.3%. No death attributable to malaria was reported by the malaria control programme during the study period.

For the purpose of the study, villages under Amoda Community Health Centre (CHC) were selected. CHC Amoda provides health services to a population of about 0.13 million. The CHC has 36 sections each comprising of about 4,000 population living in three to five villages. Malaria surveillance is carried out by a male and a female Multi-Purpose Health Worker (MPHW-M/F) in each section by undertaking fortnightly door-to-door visits. Surveillance in the study villages was strengthened by deploying two trained local volunteers in each of the study arm. Blood slides were prepared from febrile cases and anti-malarials were administered to all microscopically confirmed malaria positive cases as per the national drug policy. Based on previous year’s malaria incidence data, villages with >2 API qualify to receive two rounds of indoor residual spraying (IRS) of insecticide. Under the national programme in district Kanker, synthetic pyrethroids have been used for IRS since mid-1990. No IRS was undertaken in villages selected for the trial during the first phase of the study (September 2006 to August 2007) and also during 2008. However, two rounds of IRS with alpha-cypermethrin 5% WP were given in study villages with >2 API during 2009.

### Selection of study villages and distribution of nets

Village level malaria information for the year 2006 was retrieved from the CHC records and after careful consideration, 16 villages with varying degree of endemicity were identified for the study. Baseline data on malaria incidence and mosquito density were generated for three months from September to November 2006 from all villages before randomizing them into three arms, viz. Interceptor net (LN), untreated polyester net (UN) and no net. A population census was carried out in every village during the pre-intervention period and all the houses in the selected villages identified for net distribution were numbered. The census also revealed that about 1.13 nets/house were owned by the community. Interceptor nets, rectangular netting consisting of 75 denier multifilament, 100% polyester, with minimum 250 kPa bursting strength, mesh: 24 holes/cm^2^; density: 30 ± 2 g/m^2^; active ingredient: 200 mg/m^2^ alpha-cypermethrin coated on polyester fibers, warp knitting and untreated polyester nets (100% polyester, multifilament yarn: 100 denier strength with a minimum 405 kPa bursting strength, mesh: 24 holes/cm^2^; density: 30 ± 2 g/m^2^) were distributed free of cost during November 2006. A total of 932 Interceptor nets (one net per house) were distributed in five villages (population 5316; parasite incidence (PI)/1000 population-14.3); similarly, 909 untreated polyester nets were given in six villages (population 4802; PI-6.6) and another group of five villages (population 3865; PI-3.1) was kept as no net control area. Investigators ensured to cover all the target population in the villages for distribution of nets. Active surveillance was strengthened in all study villages by ensuring the adequate supply of materials with the MPHWs for better management of the disease during the trial period. Inhabitants were educated about the proper usage, storage, washing and up-keep of nets.

### Ethical approval and informed consent

The study was approved by the Ethics Committee of the National Institute of Malaria Research, New Delhi, India. Villagers were informed about the aims and objectives of the study through house-to-house contact and group meetings. The written consent of each household was obtained for his/her willingness to participate in the study before distributing the nets. Information on various aspects of the study was read in the local language for obtaining their written consent.

### Mosquito collections

Mosquito collections were made from four fixed dwelling rooms during the morning hours (0600–0800 hrs) in two villages in each of the three study arms once every month. Resting mosquitoes were caught using an aspirator and flash light for fifteen minutes each employing a trained person on mosquito collection. Employing two trained personnel, the room was then sprayed with pyrethrum solution (0.2% in kerosene) using hand held atomizers and after 15 min all mosquitoes found dead on the floor-sheets were collected and transferred into paper cups with moist cotton pad at the bottom to prevent desiccation. Mosquito species were identified and their abdominal condition was recorded. Unfed *An. culicifacies* were dissected for parity using the WHO procedure [12]. Baseline mosquito collection was carried out for three months before setting up sentinel rooms for organizing routine monthly monitoring. WHO tube test using 0.1% alpha-cypermethrin impregnated papers (diagnostic dose) was conducted to determine the susceptibility of wild caught *An. culicifacies* in the beginning of the study.

### Assessment of usage pattern, net washing behaviour and physical aspects of nets

Using a structured pre-tested questionnaire, adult households were interviewed to assess the net usage pattern, net washing behaviour and physical integrity of the net. In all, four surveys were carried out at six-monthly intervals at 18, 24, 30 and 36 months post-net distribution in Interceptor villages. In the first three surveys, questionnaire was administered to about 10% of the households selected randomly. Houses once surveyed were excluded from the subsequent surveys. However, in the survey carried out after three years of net distribution, all houses which were initially given the Interceptor LN were surveyed. The houses were checked for physical possession of the nets.

All the nets were examined for the presence, size (by measuring diameter in cm) and location of holes, tears, burns, open seams, stitches and knots. Based on the size, holes were grouped into four categories, viz. Size 1: smaller than a thumb (0.5–2 cm), Size 2: larger than a thumb but smaller than a fist (2–10 cm), Size 3: larger than a fist but smaller than a head (10–25 cm) and Size 4: larger than a head (>25 cm) and respectively weighed as 1, 23, 196 and 576 [13]. Hole index for each net was calculated as: (1 × no. of size − 1holes) + (23 × no. of size − 2holes) + (196 × no. of size − 3holes) + (576 × no. of size − 4holes).

### Wash resistance and bio-efficacy of interceptor LNs

Four Interceptor LNs and two untreated nets were washed 20 times in the laboratory. Nets were washed separately and individually in plastic tubs. A teaspoon full of Surf Excel® (washing powder, M/s. Hindustan Unilever Ltd, Mumbai, India) was dissolved in five litres of tap water (26 ± 2°C) and nets were rinsed four to five times and, thereafter two to three times in clean water before hanging on laundry line under shade for drying for 24 h. Nets were stored under the optimal room temperature and humidity between the washes. Bioassays were conducted after two to three days post washing. On each net a cone was fixed and held in place using a rubber band. Laboratory-reared *An. culicifacies* mosquitoes (sugar fed, 2–5 day old), susceptible to alpha-cypermethrin were introduced into each cone and exposed for 3 min [14]. This was repeated twice on each of the four cones fixed on four nets. Thus, a total of 60 and 30 mosquitoes were exposed on Interceptor LNs and untreated polyester nets respectively. Number of mosquitoes knocked-down after one hour and mortality after 24 hour of exposure were recorded. The WHO criteria for efficacy is ≥95% knock-down after one hour and ≥80% mortality after 24 hour. Abbott’s formula was applied for correcting the mortality in test replicates if the mortality in control replicates was between 5 and 20%. If the mortality in control replicates was >20%, the tests were discarded.

### Sampling for chemical analysis

After 36 months of household use in the field, 30 Interceptor LNs were retrieved from thirty households randomly selected from all the study villages. New Interceptor LN was provided in lieu of withdrawn net. From each of the 30 LNs, four pieces of 30 cm × 30 cm size were cut from positions 2 to 5 as per WHO protocol using sharp scissors. Four sub-samples were assembled as one sample, rolled up and placed in a new, clean aluminium foil, labeled and stored at +4°C prior to dispatch to Walloon Agricultural Research Centre (CRA-W), Gembloux, Belgium for chemical assay which is a WHO collaborating centre. Sample from position 1 was not cut since netting fabric at this position is subjected to excessive abrasion in routine use (this portion of net is frequently manipulated while tucking the nets under the bed/mattress). Analytical method RESSM016 of the Walloon Agricultural Research Centre (CRA-W) was used. The analytical method was already validated and is ISO 17025 accredited for alpha-cypermethrin in coated LNs. Results were expressed as g alpha-cypermethrin (cis II) / kg and converted to mg alpha-cypermethrin (cis II)/m² using the density [15].

### Statistical analysis

All the data were entered in MS Excel and statistical analysis was performed using Excel programme. Categorical variables were compared using Chi-square test and continuous discrete variables were tested using Analysis of variance (ANOVA) to test the significance among the three study areas. A p-value <0.05 was considered as significant.

## Results

Number of mosquitoes collected from Interceptor net, untreated net and no net villages are presented in Table 1. In susceptibility tests, *An. culicifacies* was found 100% susceptible to 0.1% alpha-cypermethrin. During the pre-intervention period, there was no significant difference in the number of *An. culicifacies*, other anophelines and culicine mosquitoes caught from the three study areas (p > 0.05). The proportion of blood fed females of *An. culicifacies* and culicines among all collected in the three study areas during pre-intervention period did not differ significantly (p > 0.05); while it differed significantly for other anophelines (p < 0.05).

**Table 1:** Entry rate of mosquitoes determined by pyrethrum spray collections in rooms with Interceptor LNs, untreated nets and no net

**Mosquito species**	**No. of rooms**	**Interceptor**		**Untreated net**		**No net**		**ANOVA**	**Chi-square**
		**Females / room**	**% blood fed**	**Females / room**	**% blood fed**	**Females / room**	**% blood fed**	**Females/ room**	**Blood fed**
**Pre-intervention (Sep-Nov 2006)**									
*An. culicifacies*	8	3.5	28.5	2.4	31.5	4	40.1	F_2,9_ = 0.35; P = 0.708	χ2= 1.4; p=0.55
Other anophelines	8	7.3	24.1	4.4	51.4	7.5	40	F_2,9_ = 0.40; P = 0.68	χ2= 7.24; p=0.02
Culicines	8	8	43.7	6	41.6	22.5	38.3	F_2,9_ = 0.63; p = 0.55	χ2 = 0.638; p=0.72
**Intervention (Dec 2006-Aug 2007)**									
*An. culicifacies*	36	2.9	9.6	8.5	22.4	22.9	26.6	F_2,51_ = 11.5; p = 0.0003	χ2= 15.8; p<0.0001
Other anophelines	36	26.5	5.5	123.3	21	199.3	28.1	F_2,51_ = 2; p = 0.144	χ2 =266; p=0.0001
Culicines	36	8.3	28.2	23	59.1	16.1	48	F_2,51_ = 3.42; p = 0.04	χ2 = 49.3; p=0.0001

During the intervention period, the number of *An. culicifacies* mosquitoes collected from Interceptor net villages was significantly less than that of in the untreated polyester net and no net villages (F = 11.5; p = 0.0003). Similar results were obtained for *Culex* spp. as well (F = 3.42; p < 0.05), however, the number of other anophelines caught from the three study areas did not vary significantly (p > 0.05). The proportion of blood fed females among all collected mosquitoes decreased considerably in Interceptor net villages and the results were statistically significant among the three study areas (p < 0.0001). The results clearly indicated the impact of Interceptor LNs in reducing the prevalence and blood feeding of *An. culicifacies*.

Unfed *An. culicifacies* collected from human dwellings (hand collection + pyrethrum spray collection) were dissected for parity. Parity rates during the intervention period of 9 months (December 2006 to August 2007) were 17.2% (27/157), 43.9% (58/132) and 28.2% (74/262) respectively in Interceptor net, untreated polyester net and no net villages. Parity rate of *An. culicifacies* mosquitoes in Interceptor net villages was much lower than that of the untreated net and no net villages (χ^2^ = 25.1; p < 0.0001) indicating reduction in vector longevity in the former as compared to the latter group of villages.

Malaria incidence data for the three study arms are given in Table 2. During the pre-intervention period of three months (September-November 2006), slide positivity rate (no. of malaria positive blood slides/100 slides) was 8.4, 6.9 and 3.2 in Interceptor nets, untreated polyester nets and no net villages respectively. During the intervention period of 9 months (December 2006-August 2007), SPR in the above group of villages recorded was 3.7, 6.5 and 11% respectively. Thus, there was a significant drop in the SPR in LN villages in comparison to untreated net and no net villages and the results were statistically significant (p < 0.0001). In contrast, in untreated net and no net villages, the parasite incidence (malaria incidence/1,000 population) increased in comparison to pre-intervention period. The results thus clearly demonstrated the effectiveness of Interceptor nets in reducing malaria incidence.

**Table 2:** Results of fever surveillance in villages with Interceptor LNs, untreated nets and no net during pre- intervention and intervention phases

**Attributes**	**Interceptor net**	**Untreated net**	**No Net**	**P- value**
	**(Pop. 5316)**	**(Pop. 4802)**	**(Pop. 3865)**	
**Pre-intervention (Sep-Nov 2006)**				
No. of slides collected	902	447	377	
*P. vivax*	0	5	3	
*P. falciparum*	76	26	9	
Slide Positivity Rate (SPR)	8.4	6.9	3.2	χ2=11.4; p=0.003
Parasite incidence/1000 population	14.3	6.6	3.1	
**Intervention (Dec 2006-Aug 2007)**				
No. of slides collected	2348	1836	1151	
*P. vivax*	0	17	21	
*P. falciparum*	87	102	150	
Slide Positivity Rate (SPR)	3.7	6.5	11	χ2=81.5; p=0.000
Parasite incidence/1000 population	16.4	24.8	44.2	

Results of cone bio-assays carried out in laboratory on Interceptor nets washed up to 20 times revealed 86.7% mortality in *An. culicifacies* which met the efficacy criteria for LNs set by WHO (Table 3).

**Table 3:** **Results of laboratory cone bioassays against*An. culicifacies*exposed on untreated nets and Interceptor LNs**

**No. of**	Untreated net*			**Interceptor LN**			**Corrected**
**washes**	**% 1 h knockdown**	**24 h mortality**	**% killed**	**% 1 h knockdown**	**24 h mortality**	**% killed**	**mortality (%)**
0	0	1	3.3	96.7	60	100	100
5	0	1	3.3	98.3	59	98.3	98.3
10	0	1	3.3	96.7	54	90.0	90.0
15	0	1	3.3	95.0	51	85.0	85.0
20	0	1	3.3	95.0	52	86.7	86.7

*30 and 60 mosquitoes were exposed on untreated (2) and Interceptor LNs (4) respectively.

About 33 to 40% of households reported sleeping under the LN seasonally (generally during post monsoon months) but every night in surveys carried out at various intervals (Table 4). Net usage rate (year round and every night) ranged from 26.8 to 33%. Net users comprised 12% children under <5 years and females of all age groups comprised 52.2%. More than 90% of the nets were found washed during each survey. The households reported washing >50% nets at least once in 4 months, or >80% nets in 6 months of use. Only 15.5% nets were washed once a year.

**Table 4:** Results of questionnaire based survey of households on net usage pattern and washing behaviour at different intervals after Interceptor LN distribution

**Parameters**	Months of observations*							
	**18 months**		**24 months**		**30 months**		**36 months**	
	**No.**	**%**	**No.**	**%**	**No.**	**%**	**No.**	**%**
**i) Net usage**								
- year round and every night	26	26.8	30	30.9	32	33.0	199	27.2
- year round but occasionally	15	15.5	15	15.5	23	23.7	58	8.0
- seasonally** but every night	35	36.1	39	40.2	32	33.0	299	40.9
-seasonally** but occasionally	21	21.6	13	13.4	10	10.3	175	23.9
**ii) Proportion of nets washed**								
- washed nets	89	91.8	92	94.8	89	91.8	699	95.6

*About 10% households were visited in surveys carried out after 18, 24 and 30 months of follow up while after 36 months all households were surveyed.

**Season of perceived mosquito nuisance and net usage (generally post monsoon months).

Households used as many as 13 brands of commercial soaps and soap powders for washing the nets. 97.9% nets were washed with soap powder and only 0.3% with soap cake. Physical examination of nets revealed a gradual increase in the number of nets developing holes from 19.6% at 18 months to 30.9, 60.8 and 73.7% at 24, 30 and 36 months intervals respectively (Table 5). A gradual increase was observed in the number of holes from 2.7/net after 18 months of distribution to 7.3/net after 36 months. Similarly, a gradual increase in mean hole index from 13.2 after 18 months to 252.2 after 36 months was observed. The terminal survey of 731 available nets revealed that majority of holes was on the sides and on the lower half of the net. A total number of 750 repairs were found on the surveyed nets (1.02 repairs/net) of which 479 (63.9%) were stitches while 269 (35.8%) and only 2 (0.3%) were knots and patches respectively. In the surveys carried out after 18, 24 and 30 months the total number of repairs in the form of stitches, knots and patches were 3, 9 and 22 respectively. The repairs were necessitated mainly due to open seams, burns and rat biting.

**Table 5:** Physical aspects of Interceptor LNs observed at different interval of distribution*

**Parameters**	**Months of observation post-net distribution**			
	**18**	**24**	**30**	**36**
**No. of nets surveyed**	97 (10%)	97 (10%)	97 (10%)	731 (78.4%)
**Proportion with any holes**	19.6%	30.9%	60.8%	73.7%
**Average no. of holes/net**	2.7 (52/19)	3.0 (91/30)	3.7 (221/59)	7.3 (3933/539)
-size 1	1.5	1.06	1.5	5.1
-size 2	1.1	1.3	1.9	1.2
-size 3	0.05	0.7	0.2	0.9
-size 4	0.05	0.03	0.1	0.07
**Mean hole index**	13.2	55.7	87.1	252.2
**Proportion of holes/net**				
-lower half	78.6	89.6	72.9	61.6
-upper half	14.3	6.9	21.6	24.6
-roof	7.1	3.5	5.5	13.8
**Repairs/net**				
-stitches	0.01	0.07	0.23	0.7
-knots	0.02	0.01	0	0.4
-patches	0	0.01	0	0.003
**Aspect of net**				
-clean	56.7	58.8	41.2	42.5
-a bit dirty	0	0	0	29.3
-dirty	41.2	41.2	58.8	19.6
-very dirty	2.1	0	0	8.6

*A total of 932 Interceptor LNs were distributed (at the rate of one net per house) in November 2006.

Proportion of nets found to be clean, a bit dirty, dirty and very dirty were 42.5, 29.3, 19.6 and 8.6%, respectively. The terminal survey revealed that 731 (78.4%) out of 932 nets distributed initially were found in possession of the households, 169 nets were found missing (an attrition rate of 18.1%) and 32 houses (3.2%) were found locked at the time of survey. Assuming an equal attrition rate of 18.1% in locked houses, then the overall percentage of nets available with the community after three years of distribution would come to 81.2%.

Results of chemical analysis revealed that 30 LNs withdrawn after three years of household use had mean alpha-cypermethrin (a.i.) content of 43.5 mg a.i./m^2^ (95% CI: 31.2, 55.8) which is equivalent to 1.24 g a.i./m^2^ (0.9, 1.58). Thus, there was a reduction of about 78% in alpha-cypermethrin content from the baseline specification of 200 mg/m^2^ in nets under household use after three years.

## Discussion

Use of long lasting insecticidal nets is an effective intervention tool for protection from malaria. The present study showed that usage of nets reduced the feeding success rate of vector as well as other nuisance mosquitoes. Blood feeding inhibition was highest in Interceptor villages, which may be attributed to excito-repellent action of treated nets. There was a reduction in the vector density in Interceptor LN villages as compared to untreated and no net villages.

There was a decline in malaria incidence rate in villages with Interceptor nets during the intervention period as compared with that during the pre-intervention period and also in comparison to untreated and without net villages. Similar results were found in studies carried out in India and elsewhere [4-8].

In the present investigation, >80% mortality in *An. culicifacies* was reported in 20 times washed Interceptor nets, which is in agreement with the WHO criteria [14]. Compliance of use of nets every night was observed to be >60% in surveys carried out at different study intervals. In studies carried out in Uganda, 86% of Interceptor LNs were being regularly used by inhabitants even after three years [16]. The comparative lower usage found in the present study may be due to prevailing hot weather conditions during most parts of the year, except during the colder months from November to February.

The frequency of washing of nets ranged from one wash per year in 16% of households to 24 washes per year in about 4% of households. More than 80% of LNs were found washed in surveys carried out at different study intervals. In each survey about 30% of nets were found to be washed at six monthly intervals. As many as 13 brands of local soaps and soap powders were used by the villagers for washing the nets.

The households are habitual to sweep the house floor daily and burn jungle wood for cooking resulting in settling of dirt and soot over the nets. More so, if the kitchen is in proximity of the room in which the net is used. The reason for more frequent washes of nets in certain houses was related with the dirty looks given by the net which is considered to be unhealthy and socially unacceptable as it has been reported in studies carried out in urban Dar-es-Salaam [17].

The continuous use of nets resulted in the wear and tear. A gradual increase in the proportion of nets with any type of holes showed an increasing trend from 19.6% after 18 months to 73.7% after three years, which is in agreement with results obtained from western Uganda [16]. Major causes of wear and tear of the nets were due to rough handling, frequent washing, rat biting, damage caused by cats and occasionally chewing by cattle. In spite of all these adversaries, about 81% households had retained the nets after three years of distribution although a gradual increase in the hole index from 13.2 after 18 months to 252.2 after three years was observed. We did not find any net which was kept as a rag and not being used by the households during any of the surveys carried out after 18, 24, 30 and 36 months of net distribution. As a result of this the attrition rate largely remained unaffected.

Mean alpha-cypermethrin residue on the LNs after three years of household use showed a reduction of about 78% from the baseline specification, but still retaining a mean content of 43.5 mg/m^2^. Similar observations have been reported from western Uganda [16].

This study reports the effectiveness and durability of LN in a malaria endemic tribal dominated forested rural area in Indian sub-continent. LN as an intervention tool in this area is still in its infancy of implementation. The results of the present study are promising and therefore an intervention based on LN is strongly recommended in areas similar to the present one. This is based on the findings that tribal populations mud-plaster their houses half a dozen times during the year, which is done for the proper upkeep of mud-walled house. Under such situations, IRS with highly effective insecticide is unlikely to obtain desired epidemiological impact.

## Conclusions

The Interceptor LNs were found to be effective in controlling the density of malaria vector *An. culicifacies* to a great extent. A significant decline in the overall entry rate of mosquitoes in LN houses was observed. Number of blood fed mosquitoes decreased considerably in LN houses and the results were found statistically significant on this aspect of the study. A decline in slide positivity rate occurred in the Interceptor net villages as compared to untreated polyester net and no net villages. Despite the short duration of the study parasite incidence in the LN villages remained comparable during the pre-intervention and post–intervention periods. However, the above indicator showed an increasing trend in untreated net and no net villages.

Interceptor LNs retained effective bio-efficacy causing >80% mortality in *An. culicifacies* and withstood 20 laboratory washes using a locally available detergent powder. The community compliance and acceptance was high and no side effects were reported during the entire course of the study. The users required better awareness about the upkeep of nets and washing practices. With very high retention rate (survivorship), physical condition of the nets (fabric integrity) and an availability of effective insecticide content it can be safely said that Interceptor LNs have a durability of three years. Thus the duration of an effective intervention based on Interceptor LN can safely be estimated to be of about three years under the environmental conditions prevailing in central part of India.

## Competing interest

The authors declare that they have no competing interests.

## Authors’ contributions

BRM and SSN contributed to the planning, execution and supervision of laboratory and field activities, data analysis and drafting the manuscript, US performed the statistical analysis and contributed in preparing the first draft of the manuscript, DAP and KR planned the study and contributed in data interpretation and finalizing the manuscript. All authors contributed equally in preparing the final version of the text and have read and agreed to the manuscript.
